# Association of Neighborhood Alcohol Environment With Alcohol Intake and Cardiovascular Risk Factors in India: Cross-Sectional Evidence From APCAPS

**DOI:** 10.3389/fcvm.2022.844086

**Published:** 2022-04-29

**Authors:** Yingjun Li, Poppy Alice Carson Mallinson, Aastha Aggarwal, Bharati Kulkarni, Sanjay Kinra

**Affiliations:** ^1^Department of Epidemiology and Health Statistics, Hangzhou Medical College School of Public Health, Hangzhou, China; ^2^Department of Non-communicable Disease Epidemiology, London School of Hygiene and Tropical Medicine, London, United Kingdom; ^3^Centre for Chronic Conditions and Injuries, Public Health Foundation of India, New Delhi, India; ^4^National Institute of Nutrition, Hyderabad, India

**Keywords:** alcohol environment, availability, accessibility, alcohol intake, cardiovascular risk, APCAPS

## Abstract

There are more and more proofs about the impact of neighborhood alcohol environment on alcohol-associated events. The relationship between the neighborhood availability and accessibility of alcohol outlet with individual level of alcohol consumption along with 11 cardiovascular risk factors was explored for the first time in India using data from the 3rd follow-up of the Andhra Pradesh children and parents study (APCAPS) (*n* = 6156, for liquor intake and 5,641 for heart and blood vessel risk elements). In fully adjusted models, volunteers in the lowest tertile performed worse than volunteers in the highest tertile of distance to the closest alcohol outlet were more probably to exhibit less alcohol consumption (−14.40 g/day, 95% *CI*: −26.21, −2.59). A unit per km^2^ rise in alcohol outlet density in 400 m buffering area was related to a rise in waist circumference (1.45 mm, 95% *CI*: 0.13, 2.77), SBP (0.29 mmHg, 95% *CI*: 0.09, 0.49), and DBP (0.19 mmHg, 95% *CI*: 0.03, 0.35). A unit per 100 m rise in distance to the closest alcohol outlet was related to a rise in waist circumference (−2.39 mm, 95% *CI*: −4.18, −0.59), SBP (−0.41 mmHg, 95% *CI*: −0.68, −0.15), and DBP (−0.29 mmHg, 95% *CI*: −0.51, −0.07). Neighborhood availability of alcohol outlets within immediate locality of participants’ households had a closer relationship with cardiovascular risk factors than that within the whole village. Public health policies designed to limit neighborhood availability and accessibility of alcohol outlets ought to be advocated in southern India.

## Introduction

Cardiovascular disease (CVD) causes huge disease burden and deaths worldwide (1). In India, it contributed 28.1% of the total deaths and 14.1% of the total disability-adjusted life-years in 2016 (2). Alcohol use is a key modifiable risk factor for acute and chronic diseases globally, causing 2.8 million deaths in 2016 (3). Heavy drinking is associated with an increased risk of initial presentation of several symptomatic manifestations of CVD, such as coronary death, heart failure, cardiac arrest, transient ischemic attack, ischemic stroke, intracerebral hemorrhage, and peripheral arterial disease (4). In India, alcohol attributable deaths would lead to a loss of 258 million life years between 2011 and 2,050, causing significant negative health impact and economic burden (5).

Data from multiple countries indicate a close connection between the neighborhood alcohol environment, especially the availability and accessibility of alcohol (on and/or off-premises), and the individual alcohol consumption (6). However, most of the existing studies have been conducted in high-income countries and have focused on the association between alcohol environment and drinking behaviors and beliefs among adolescents or young adults (7–9). Very few studies have been carried out in low- and middle-income countries. Ibitoye et al. found that high density of alcohol-selling outlets and outdoor advertisements increased alcohol use among adolescents in Tanzania (10). Study from rural South Africa found that density of alcohol outlets was associated with problematic drinking among young men (11).

Further, there is increasing evidence about the impact of neighborhood alcohol environment on alcohol-related events including experienced violence, injuries, and drunk-driving fatalities (12–14). Higher alcohol outlet density was also shown to be associated with non-injury health outcomes such as liver problems, mental health, sexually transmitted infections, and total deaths (15–17). By now, to the best of our knowledge, no study has addressed the association between the availability and accessibility of alcohol with individual level of alcohol consumption and cardiovascular risk factors in general population in low- and middle-income countries.

In order to inform policies for the control of local alcohol use and disease burden of CVD in low- and middle- income countries, further evidence on the effect of alcohol environment on individual alcohol use as well as cardiovascular risk is urgently needed. Therefore, we carried out data analysis from a peri-household cohort study in southern India here to investigate whether access to and availability of alcohol outlets in the local neighborhood is related with alcohol consumption and cardiovascular risk factors.

## Materials and Methods

### Study Participants

The Andhra Pradesh children and parents study (APCAPS) study has been explained previously (18, 19). In short, it’s an inter-generation cohort in south India, which started with the follow-up of the participants of the Hyderabad Nutrition Trial (HNT) conducted from 1987 to 1990. It was conducted in 29 villages close to Hyderabad in Ranga Reddy district, Andhra Pradesh. The data used in this cross-sectional analysis are from the 3rd follow-up where the index participants of HNT and their family members were enrolled. Between 2010 and 2012, data on sociodemographic features, lifestyle-related factors, anthropometric measurements, and heart and blood vessel biomarkers of study participants was collected (20–22). Of the 6,659 participants from 3rd follow-up, current analysis is based on data from 6,156 participants after removing 503 participants with missing information on household address, sociodemographic features, or drinking quantity. For analysis of association between alcohol environment and cardiovascular risk factors, data from *n* = 5,641 participants was considered after removing participants with diagnosed heart disease and/or missing data on lifestyle risk factors.

### Global Positioning System-Dependent Surveys of Alcohol Environment

In 2016, geographic coordinates (latitude and longitude) of all the alcohol outlets in study villages were captured by Global Positioning System (GPS). Both availability and accessibility were examined to measure neighborhood alcohol environment. Availability of alcohol outlets was measured near the participants’ households and in the entire village by determining density of outlets in two buffering regions of participants’ households, 400 and 1,600 m, respectively. The degree of accessibility of an alcohol outlet was measured as the distance from a household to the closest outlet. The R software 3.5.1 was adopted to obtain the geography exposures.

### Alcohol Intake and Cardiovascular Risk Factors

Data on alcohol intake were collected through questionnaire by well-experienced researchers in the 3rd follow-up. Fasting glucose, insulin, body mass index (BMI), waist circumference, waist-hip-ratio (WHR), blood pressure, serum triglycerides, total cholesterol, high-density lipoprotein cholesterol (HDL-C), and low-density lipoprotein cholesterol (LDL-C) were analyzed as cardiovascular risk factors herein. Fasting blood specimens were collected and prepared for physiological measurements and biochemical assays in the 3rd follow-up (19). Glucose level was estimated *via* the glucose oxidase/peroxidase-4-aminophenazone-phenol procedure. Insulin level was estimated using an electrochemiluminescence immunoassay. Concentrations of triglycerides and cholesterols (HDL, total) were measured using enzymatic calorimetric method (23, 24). LDL-C level was calculated by standard Friedewald–Fredrickson equation (25). The anthropometry evaluations included weight (identified to the nearest 0.1 kg by digitalized SECA scale), height (nearest 1 mm by portable plastic stadiometer), as well as waist circumference and hip circumference (nearest 1 mm by portable plastic stadiometer). The BMI was calculated by dividing weight in kilograms by the square of height in meters. The WHR was calculated by dividing waist circumference by hip circumference. The blood pressure was measured at the right arm in the seated posture *via* an oscillometry equipment (Omron M5-I). Three results were recorded and the average value was considered for these measurements.

### Covariates

The data on sociodemographic and lifestyle variables such as age, gender, educational level (illiterate, primary school, middle school, and above), occupation (unskilled, skilled, and others), Standard of Living Index (SLI) (low, middle, and high), smoking (never, former, and current), drinking (gram/day), and physical activity (extremely inactive, sedentary, moderately active, and vigorously active) were collected using standardized questionnaires by practiced researchers. The SLI was adopted to measure the family socioeconomic conditions (20). The data on diet intake (in the last year) and physical activities (in the last week) were gathered by semi-quantitation questionnaires (19).

### Statistical Analysis

Considering the possible clustering of neighborhood alcohol environment, three-level mixed-effects linear regression was employed to evaluate the relationship between the densities and distances of alcohol outlets (exposures) with individual level of alcohol consumption (outcome 1) and cardiovascular risk factors (outcome 2). In the analysis of alcohol consumption, age (continuous, year), and gender [categorical, (male and female)] were adjusted in model 1; education level [categorical, (illiterate, primary school, and middle school and above)], occupation [categorical, (unskilled, skilled, and others)], and SLI [categorical, (low, middle, and high)] were further adjusted in model 2. In the analysis of cardiovascular risk factors, we adjusted for same variables in model 1 and 2; we further included tobacco use [categorical, (never, former, and current)], physical activity [categorical, (extremely inactive, sedentary, moderately active, and vigorously active)], and daily energy intake (continuous, kcal/day) in model 3. To account for the effect of disease diagnosis on behaviors, we performed a sensitivity analysis by excluding participants with diagnosed high blood pressure and/or diabetic disease to account for the effect of disease diagnosis on behaviors.

Stata software 15.1 was used for statistical analyses. PASS software 15.0 was used for power analysis in linear regression. Take SBP for example, a sample size of 5,641 achieves 99% power to detect a change in slope from 0.00 under the null hypothesis to 0.31 under the alternative hypothesis when the standard deviation of the alcohol outlet density (units/km^2^) within 400 m buffering area is 2.96, the standard deviation of SBP (mmHg) is 15.89, and the two-sided significance level is 0.05.

## Results

### Baseline Characteristics

Our data set for the analysis of the association between alcohol environment and cardiovascular risk factors included 5,641 individuals (3,244 men and 2,397 women) residing in 29 villages with median age of 29.1 years. Table 1 presents the sociodemographic characteristics of participants, densities and distances of alcohol outlets, and measures of alcohol intake and cardiovascular risk factors. The median intake of alcohol was 29.1 gram (interquartile range: 68.6 gram) per day and the densities of alcohol outlets (on and/or off-premise) within 400 m were higher than that within 1,600 m.

**TABLE 1:** Socio-demographic, food environment, and biological characteristics of APCAPS participants.

Characteristics (*N* = 5,641)	All	
	Median	P5-P95
**Socio-demographic factors**		
Age (years)	29.1	16.2–60.0
**Sex (%)**		
Male	57.5	
Female	42.5	
**Education (%)**		
Illiterate	35.7	
Primary school	23.3	
Middle school and above	41.0	
**Occupation (%)**		
Unskilled	41.32	
Skilled	27.83	
Others	30.85	
**Standard of living index (%)**		
Low	27.3	
Middle	37.6	
High	35.2	
Alcohol drinking (g/day)	29.1	0–373.6
Energy intake (kcal/day)	2117.3	1106.2–3987.4
**Smoking status (%)**		
Never	74.8	
Former	1.1	
Current	24.1	
**Physical activity (%)**		
Extremely inactive	15.2	
Sedentary	54.7	
Moderately active	25.1	
Vigorously active	5.0	
Alcohol environment		
**Alcoholoutlet density (units/km^2^)**		
≤400 m	4.0	0–9.9
≤1,600 m	0.4	0.1–1.0
Distance to the nearest outlet(m)	159.8	31.0–505.8
**Biological characteristics**		
BMI (Kg/m^2^)	20.0	15.3–27.4
Waist circumference (mm)	705.0	563.0–905.5
WHR	0.8	0.7–1.0
Systolic blood pressure (mmHg)	117.3	99.7–150.0
Diastolic blood pressure (mmHg)	76.7	59.3–101.3
Fasting glucose (mg/dl)	90.9	75.5–118.1
Insulin (Uu/ml)	5.2	1.1–16.4
Triglycerides (mmol/L)	1.2	0.6–3.1
Total cholesterol (mmol/L)	4.2	2.8–6.04
HDL cholesterol (mmol/L)	1.1	0.7–1.7
LDL cholesterol (mmol/L)	2.4	1.3–3.9

*P5, 5th percentile; P95, 95th percentile.*

### Alcohol Outlet and Alcohol Consumption

Table 2 presents associations between availability and accessibility of alcohol outlets and individual level of alcohol consumption. In model 1, compared with participants at the lowest tertile, those at the highest tertile of distance to the nearest alcohol outlet were more likely to have lower level of alcohol consumption [−14.07 g/day, 95% *CI*: −26.02, −2.12]. The association stayed robust in fully adjusted model 2 (−14.40 g/day, 95% *CI*: −26.21, −2.59). There was no association between density of alcohol outlet within 400 m buffering area or 1,600 m buffering area with daily alcohol intake.

**TABLE 2:** Association between alcohol availability and accessibility with individual level of alcohol intake in APCAPS participants (*n* = 6,156).

	Model 1			Model 2		
	β	95% *CI*	*p*	β	95% *CI*	*p*
**Outlet density in 400 m buffer (units/km^2^)**						
Linear, per 1- units/km^2^ increment	0.06	−1.97, 2.09	0.956	0.14	−1.87, 2.14	0.893
Tertile 1	Reference			Reference		
Tertile 2	6.72	−8.76, 22.21	0.395	8.13	−7.16, 23.43	0.297
Tertile 3	4.26	−13.20, 21.71	0.633	5.50	−11.77, 22.76	0.533
**Outlet density in 1,600 m buffer (units/km^2^)**						
Linear, per 1- units/km^2^ increment	−25.19	−56.15, 5.77	0.111	−23.76	−54.30, 6.77	0.127
Tertile 1	Reference			Reference		
Tertile 2	1.85	−21.11, 24.80	0.875	3.21	−19.41, 25.82	0.781
Tertile 3	−11.73	−32.06, 8.60	0.258	−10.69	−30.73, 9.35	0.296
Distance to the nearest outlet						
Linear, per 100 m increment	−0.75	−3.35, 1.86	0.574	−0.91	−3.48, 1.67	0.490
Tertile 1	Reference			Reference		
Tertile 2	−5.01	−15.88, 5.85	0.366	−4.23	−14.95, 6.50	0.440
Tertile 3	−**14.07**	−**26.02,**−**2.12**	**0.021**	−**14.40**	−**26.21,**−**2.59**	**0.017**

*Model 1 is adjusted for age and sex. Model 2 is adjusted for model 1 + education, occupation, and Standard of Living Index.*

*Bold number indicates that the estimates reach statistical significance.*

### Alcohol Outlet and Cardiovascular Risk Factors

The association between availability and accessibility of alcohol outlets and cardiovascular risk factors is presented in Table 3. In fully adjusted models, a unit per km^2^ increase in alcohol outlets’ density within 400 m buffering area was associated with an increase in waist circumference (1.45 mm, 95% *CI*: 0.13, 2.77), systolic blood pressure (SBP) (0.29 mmHg, 95% *CI*: 0.09, 0.49), and diastolic blood pressure (DBP) (0.19 mmHg, 95% *CI*: 0.03, 0.35). There was no association between densities of alcohol outlets within 1,600 m buffering area with cardiovascular risk factors.

**TABLE 3:** Association between alcohol availability and accessibility with cardiovascular risk factors in APCAPS participants (*n* = 5,641).

	Model 1			Model 2			Model 3		
	β	95% *CI*	*p*	β	95% *CI*	*p*	B	95% *CI*	*p*
**Glucose**									
**Alcohol outlet density (units/km^2^)**									
≤400 m	0.09	−0.20, 0.37	0.557	0.05	−0.24, 0.34	0.741	0.06	−0.22, 0.34	0.684
≤1600 m	2.46	−1.74, 6.67	0.251	2.29	−1.90, 6.47	0.284	2.36	−1.67, 6.38	0.251
Distanceto the nearest outlet (100 m)	−0.18	−0.57, 0.21	0.372	−0.14	−0.53, 0.25	0.472	−0.15	−0.54, 0.24	0.454
**Insulin**									
**Alcohol outlet density (units/km^2^)**									
≤400 m	0.08	−0.01, 0.18	0.095	0.05	−0.05, 0.14	0.328	0.04	−0.05, 0.14	0.380
≤1600 m	1.05	−0.38, 2.49	0.151	0.91	−0.52, 2.33	0.213	0.90	−0.54, 2.34	0.220
Distance to the nearest outlet (100 m)	−0.73	−1.63, 0.16	0.109	−0.47	−1.36, 0.41	0.296	−0.46	−1.34, 0.43	0.312
**BMI**									
**Alcohol outlet density (units/km^2^)**									
≤400 m	**0.06**	**0.01, 0.11**	**0.025**	0.04	−0.01, 0.09	0.096	0.04	−0.01, 0.09	0.130
≤1600 m	0.48	−0.21, 1.18	0.171	0.47	−0.20, 1.15	0.171	0.46	−0.24, 1.15	0.197
Distance to the nearest outlet (100 m)	−**0.08**	−**0.16,**−**0.01**	**0.021**	−0.07	−0.14, 0.00	0.067	−0.06	−0.13, 0.01	0.076
**Waist circumference**									
**Alcohol outlet density (units/km^2^)**									
≤400 m	**2.03**	**0.68, 3.38**	**0.003**	**1.53**	**0.23, 2.84**	**0.021**	**1.45**	**0.13, 2.77**	**0.031**
≤1,600 m	13.92	−4.50, 32.35	0.139	14.50	−3.15, 32.14	0.107	13.91	−4.20, 32.02	0.132
Distance to the nearest outlet (100 m)	−**2.92**	−**4.77,**−**1.08**	**0.002**	−**2.44**	−**4.23,**−**0.64**	**0.008**	−**2.39**	−**4.18,**−**0.59**	**0.009**
WHR*									
**Alcohol outlet density (units/km^2^)**									
≤400 m	0.61	−0.22, 1.45	0.152	0.51	−0.31, 1.34	0.223	0.48	−0.34, 1.31	0.251
≤1,600 m	10.87	−1.52, 23.27	0.086	11.27	−0.84, 23.38	0.068	11.20	−0.99, 23.40	0.072
Distance to the nearest outlet (100 m)	−0.68	−1.81, 0.44	0.232	−0.68	−1.78, 0.43	0.230	−0.66	−1.76, 0.44	0.241
**Systolic blood pressure**									
**Alcohol outlet density (units/km^2^)**									
≤400 m	**0.31**	**0.11, 0.51**	**0.002**	**0.29**	**0.09, 0.49**	**0.004**	**0.29**	**0.09, 0.49**	**0.005**
≤1600 m	2.74	−0.26, 5.74	0.074	2.75	−0.23, 5.74	0.071	2.76	−0.24, 5.75	0.071
Distanceto the nearest outlet (100 m)	−**0.44**	−**0.71,**−**0.17**	**0.001**	−**0.42**	−**0.68,**−**0.15**	**0.002**	−**0.41**	−**0.68,**−**0.15**	**0.002**
**Diastolic blood pressure**									
**Alcohol outlet density (units/km^2^)**									
≤400 m	**0.22**	**0.05, 0.38**	**0.009**	**0.20**	**0.03, 0.36**	**0.018**	**0.19**	**0.03, 0.35**	**0.020**
≤1,600 m	2.20	−0.20, 4.59	0.072	2.27	−0.12, 4.67	0.063	2.28	−0.12, 4.68	0.063
Distanceto the nearest outlet (100 m)	−**0.31**	−**0.53,**−**0.09**	**0.006**	−**0.29**	−**0.51,**−**0.07**	**0.009**	−**0.29**	−**0.51,**−**0.07**	**0.010**
**Triglycerides**									
**Alcohol outlet density (units/km^2^)**									
≤400 m	−0.00	−0.01, 0.01	0.757	−0.00	−0.02, 0.01	0.658	−0.00	−0.02, 0.01	0.618
≤1,600 m	0.02	−0.14, 0.19	0.780	0.04	−0.13, 0.21	0.674	0.04	−0.13, 0.21	0.679
Distanceto the nearest outlet (100 m)	0.00	−0.01, 0.02	0.719	0.00	−0.01, 0.02	0.725	0.00	−0.01, 0.02	0.711
Total cholesterol									
Alcohol outlet density (units/km^2^)									
≤400 m	−0.01	−0.02, 0.01	0.292	−0.01	−0.03, 0.00	0.174	−0.01	−0.03, 0.00	0.154
≤1,600 m	−0.03	−0.26, 0.21	0.835	−0.02	−0.26, 0.21	0.842	−0.02	−0.26, 0.21	0.849
Distanceto the nearest outlet (100 m)	−0.00	−0.02, 0.01	0.664	−0.00	−0.02, 0.02	0.792	−0.00	−0.02, 0.02	0.809
**HDL cholesterol**									
**Alcohol outlet density (units/km^2^)**									
≤400 m	−0.00	−0.01, 0.00	0.716	−0.00	−0.01, 0.00	0.878	−0.00	−0.01, 0.00	0.886
≤1,600 m	−0.07	−0.15, 0.02	0.118	−0.07	−0.15, 0.02	0.110	−0.07	−0.15, 0.02	0.110
Distanceto the nearest outlet (100 m)	0.00	−0.00, 0.01	0.497	0.00	−0.01, 0.01	0.701	0.00	−0.01, 0.01	0.704
**LDL cholesterol**									
**Alcohol outlet density (units/km^2^)**									
≤400 m	−0.00	−0.02, 0.01	0.599	−0.01	−0.02, 0.01	0.354	−0.01	−0.02, 0.01	0.326
≤1600 m	0.03	−0.16, 0.22	0.766	0.03	−0.16, 0.22	0.775	0.03	−0.16, 0.22	0.777
Distanceto the nearest outlet (100 m)	−0.01	−0.02, 0.01	0.334	−0.01	−0.02, 0.01	0.523	−0.01	−0.02, 0.01	0.535

*Independent variable multiplies 1,000 to show more information. Model 1 is adjusted for age, sex. Model 2 is adjusted for model 1 + education, occupation, and Standard of Living Index. Model 3 is adjusted for model 2 + tobacco, physical activity, and energy.

*Bold number indicates that the estimates reach statistical significance.*

In fully adjusted models, a unit per 100 m increase in distance to the closest alcohol outlet was associated with a decrease in waist circumference (−2.39 mm, 95% *CI*: −4.18, −0.59), SBP (−0.41 mmHg, 95% *CI*: −0.68, −0.15), and DBP (−0.29 mmHg, 95% *CI*: −0.51, −0.07). Association between density and distance of alcohol outlet with other cardiovascular risk factors was weak and inconsistent across different models.

### Sensitivity Analysis

Supplementary Table 1 presents associations between availability and accessibility of alcohol outlets and cardiovascular risk factors after excluding participants with high blood pressure and diabetic disease (*n* = 5,294). The results were largely unchanged except the association between alcohol outlets density within 400 m buffering area and waist circumference. The significant association disappeared in fully adjusted model (1.15 mm, 95% *CI*: −0.12, 2.43).

## Discussion

We examined associations between neighborhood alcohol environment with individual level alcohol intake and cardiovascular risk factors in data from the 3rd wave of follow-up of APCAPS. Greater distance to the nearest alcohol outlet was associated with a lower daily alcohol intake, waist circumference and blood pressure. Higher density of alcohol outlets within 400 m buffering area was associated with higher waist circumference and blood pressure. There was no link found between alcohol outlets within 1,600 m buffering zone and daily alcohol intake and cardiovascular risk factors. To the best of our knowledge, this is the first study to examine both availability and accessibility of alcohol outlets in the local neighborhood in relation to both alcohol intake and multiple cardiovascular risk factors in India.

Previous studies have indicated that density of alcohol outlets is significantly associated with increased alcohol consumption in developed countries (26–28). Few studies have been reported from the low- and middle-income countries. A study carried out in rural South Africa found that alcohol outlet density was not associated with prevalence of heavy drinking (11), which was in accordance with our results. We found no association between density of alcohol outlets within 400 m buffering area or 1,600 m buffering area with daily alcohol intake. Azar et al. found significant association between density of alcohol outlets and clubs with risky drinking only existed among residents in urban communities but not in rural areas of Australia (29). On the other hand, a systematic review found that density measures are far more frequently examined than distance measures in studies on alcohol environment and alcohol consumption (30). No significant association was found between the physical distance from an individual’s home to their nearest alcohol outlet with frequency and quantity of alcohol consumption in both urban and mixed communities of developed countries (31–33). However, a study carried out in Tanzania found that close distance to alcohol outlets may facilitate and increase adolescent alcohol use (10). In the current study, greater distance to the nearest alcohol outlet was associated with a decrease in daily alcohol consumption. The relationship between neighborhood alcohol outlet density and distance with alcohol consumption is complex and may vary due to differences in neighborhood design, travel patterns, local alcohol control policies, and enforcement, as well as drinking norms (34).

In a previous study carried out in south India, Li et al. found that higher density of unhealthy food vendors was associated with increased prevalence of obesity-related outcomes and blood pressure (35). Immoderate consumption of alcohol, like unhealthy food, is associated with serious health problems (36). To our knowledge, no study has been reported to assess the association between densities or physical distance of alcohol outlets with individual levels of cardiovascular risk factors, such as adiposity measures, glucose-insulin, blood pressure, and lipid profile. We found that not only density of alcohol outlets but also distance to the nearest alcohol outlet were associated with waist circumference and blood pressure. Annual gain in waist circumference was associated with increased consumption of alcoholic beverages in the PREDIMED trial (37). A systematic review and meta-analysis found that a reduction in alcohol intake was associated with increased blood pressure reduction in people who drank more than two drinks per day (38). Higher density and lower physical distance may increase the frequency of cues related to drinking and make alcohol more accessible, thus increasing the risk of obesity and high blood pressure through immoderate alcohol consumption.

Some limitations of this study must be acknowledged. One thing is that the coordinates of alcohol outlets were derived in 2016, which was 4 years after the third wave of follow-up in APCAPS. The temporal association between alcohol environment with higher levels of waist circumference and blood pressure cannot be ascertained in the present study. Awareness of having CVDs may change the behavior of alcohol access and alcohol drinking in participants and generate bias (39). Thus, we conducted sensitivity analysis in dataset after excluding participants with diagnosed hypertension and diabetes. The results were largely unchanged. Other limitations should also be mentioned. We only assessed the physical availability and accessibility of alcohol outlets. The available data did not allow us to look into other alcohol environment factors such as travel patterns, local alcohol control policies, or drinking norms, which could also influence alcohol consumption as well as cardiovascular risk factors. Moreover, the resulting data on alcohol use was self-reported through semi-quantitative questionnaires, which may have resulted in under-reporting by respondents (40).

## Conclusion

Despite limitations, our study is the first to examine the association between neighborhood availability and accessibility of alcohol outlets with alcohol consumption as well as cardiovascular risk factors in south India. Shorter distance to the nearest alcohol outlet was associated with increased alcohol consumption. Higher waist circumference and blood pressure were associated with a shorter distance to the nearest alcohol outlet as well as a higher density of alcohol outlets. Neighborhood availability of alcohol outlets within immediate locality of participants’ households had a closer relationship with cardiovascular risk factors than that within the whole village. Public health policies designed to limit neighborhood availability and accessibility of alcohol outlets should be encouraged in south India.

## Data Availability Statement

The original contributions presented in the study are included in the article/Supplementary Material, further inquiries can be directed to the corresponding author.

## Author Contributions

YL and PM: study conception and study design. AA, BK, and SK: data analysis. YL, PM, and AA: manuscript drafting. All authors have approved the submitted version and agreed to publication.

## Conflict of Interest

The authors declare that the research was conducted in the absence of any commercial or financial relationships that could be construed as a potential conflict of interest.

## Publisher’s Note

All claims expressed in this article are solely those of the authors and do not necessarily represent those of their affiliated organizations, or those of the publisher, the editors and the reviewers. Any product that may be evaluated in this article, or claim that may be made by its manufacturer, is not guaranteed or endorsed by the publisher.
