# Supplementary material for: Association of Neighborhood Alcohol Environment With Alcohol Intake and Cardiovascular Risk Factors in India: Cross-Sectional Evidence From APCAPS
Source: Front Cardiovasc Med. 2022 Apr 29;9:844086. doi: 10.3389/fcvm.2022.844086 (PMC9099288; doi:10.3389/fcvm.2022.844086)
Supplement: Supplementary file 1 [file Table_1.DOCX]

| Table S1 Association between availability and accessibility of alcohol outlet with cardiovascular risk factors in participants without diagnosed hypertension or diabetes (n=5,294) | | | | | | | | | |
| --- | --- | --- | --- | --- | --- | --- | --- | --- | --- |
|  | Model 1 | | | Model 2 | | | Model 3 | | |
|  | β | 95 % CI | *P* | β | 95 % CI | *P* | β | 95 % CI | *P* |
| **Glucose** |  |  |  |  |  |  |  |  |  |
| Alcohol outlet density (units/km^2^) |  |  |  |  |  |  |  |  |  |
| ≤400 m | -0.01 | -0.26, 0.23 | 0.916 | -0.04 | -0.29, 0.21 | 0.741 | -0.04 | -0.28, 0.21 | 0.757 |
| ≤1600 m | 0.26 | -3.42, 3.94 | 0.889 | 0.09 | -3.60, 3.78 | 0.963 | 0.17 | -3.44, 3.79 | 0.925 |
| Distance to the nearest outlet (100 m) | -0.17 | -0.50, 0.17 | 0.331 | -0.15 | -0.49, 0.18 | 0.375 | -0.15 | -0.49, 0.18 | 0.372 |
| **Insulin** |  |  |  |  |  |  |  |  |  |
| Alcohol outlet density (units/km^2^) |  |  |  |  |  |  |  |  |  |
| ≤400 m | 0.07 | -0.02, 0.16 | 0.130 | 0.04 | -0.05, 0.13 | 0.379 | 0.03 | -0.06, 0.12 | 0.454 |
| ≤1600 m | 1.26 | -0.11, 2.62 | 0.071 | 1.14 | -0.23, 2.50 | 0.104 | 1.12 | -0.26, 2.50 | 0.112 |
| Distance to the nearest outlet (100 m) | -0.71 | -1.57, 0.15 | 0.105 | -0.50 | -1.36, 0.35 | 0.247 | -0.49 | -1.34, 0.37 | 0.264 |
| **BMI** |  |  |  |  |  |  |  |  |  |
| Alcohol outlet density (units/km^2^) |  |  |  |  |  |  |  |  |  |
| ≤400 m | **0.05** | **0.00, 0.10** | **0.047** | 0.04 | -0.01, 0.08 | 0.145 | 0.03 | -0.02, 0.08 | 0.204 |
| ≤1600 m | 0.43 | -0.23, 1.10 | 0.201 | 0.46 | -0.19, 1.11 | 0.169 | 0.43 | -0.25, 1.10 | 0.214 |
| Distance to the nearest outlet (100 m) | **-0.08** | **-0.15, -0.01** | **0.030** | -0.06 | -0.13, 0.01 | 0.083 | -0.06 | -0.13, 0.01 | 0.096 |
| **Waist circumference** |  |  |  |  |  |  |  |  |  |
| Alcohol outlet density (units/km^2^) |  |  |  |  |  |  |  |  |  |
| ≤400 m | **1.66** | **0.37, 2.95** | **0.012** | **1.27** | **0.00, 2.53** | **0.050** | 1.15 | -0.12, 2.43 | 0.077 |
| ≤1600 m | 12.32 | -5.57, 30.20 | 0.177 | 13.67 | -3.64, 30.99 | 0.122 | 12.73 | -5.08, 30.54 | 0.161 |
| Distance to the nearest outlet (100 m) | **-2.49** | **-4.28, -0.71** | **0.006** | **-2.10** | **-3.85, -0.35** | **0.019** | **-2.03** | **-3.78, -0.28** | **0.023** |
| **WHR** ^*^ |  |  |  |  |  |  |  |  |  |
| Alcohol outlet density (units/km^2^) |  |  |  |  |  |  |  |  |  |
| ≤400 m | 0.38 | -0.45, 1.21 | 0.369 | 0.33 | -0.49, 1.16 | 0.424 | 0.29 | -0.53, 1.11 | 0.488 |
| ≤1600 m | 9.49 | -2.83, 21.82 | 0.131 | 10.14 | -2.00, 22.27 | 0.102 | 9.97 | -2.27, 22.21 | 0.111 |
| Distance to the nearest outlet (100 m) | -0.44 | -1.56, 0.69 | 0.447 | -0.49 | -1.60, 0.61 | 0.384 | -0.47 | -1.57, 0.64 | 0.408 |
| **Systolic blood pressure** |  |  |  |  |  |  |  |  |  |
| Alcohol outlet density (units/km^2^) |  |  |  |  |  |  |  |  |  |
| ≤400 m | **0.26** | **0.07, 0.44** | **0.006** | **0.24** | **0.06, 0.42** | **0.010** | **0.24** | **0.05, 0.42** | **0.012** |
| ≤1600 m | 2.36 | -0.32, 5.04 | 0.084 | 2.50 | -0.17, 5.17 | 0.066 | 2.50 | -0.18, 5.18 | 0.068 |
| Distance to the nearest outlet (100 m) | **-0.41** | **-0.66, -0.16** | **0.001** | **-0.39** | **-0.64, -0.14** | **0.002** | **-0.39** | **-0.64, -0.14** | **0.002** |
| **Diastolic blood pressure** |  |  |  |  |  |  |  |  |  |
| Alcohol outlet density (units/km^2^) |  |  |  |  |  |  |  |  |  |
| ≤400 m | **0.19** | **0.04, 0.34** | **0.016** | **0.17** | **0.02, 0.33** | **0.026** | **0.17** | **0.02, 0.32** | **0.031** |
| ≤1600 m | 1.71 | -0.50, 3.91 | 0.130 | 1.86 | -0.34, 4.07 | 0.098 | 1.86 | -0.36, 4.08 | 0.101 |
| Distance to the nearest outlet (100 m) | **-0.30** | **-0.51, -0.09** | **0.006** | **-0.29** | **-0.50, -0.07** | **0.008** | **-0.28** | **-0.49, -0.07** | **0.009** |
| **Triglycerides** |  |  |  |  |  |  |  |  |  |
| Alcohol outlet density (units/km^2^) |  |  |  |  |  |  |  |  |  |
| ≤400 m | -0.00 | -0.01, 0.01 | 0.705 | -0.00 | -0.01, 0.01 | 0.636 | -0.00 | -0.02, 0.01 | 0.580 |
| ≤1600 m | 0.01 | -0.15, 0.17 | 0.909 | 0.03 | -0.14, 0.19 | 0.760 | 0.02 | -0.14, 0.19 | 0.775 |
| Distance to the nearest outlet (100 m) | 0.00 | -0.01, 0.02 | 0.646 | 0.00 | -0.01, 0.02 | 0.670 | 0.00 | -0.01, 0.02 | 0.650 |
| **Total cholesterol** |  |  |  |  |  |  |  |  |  |
| Alcohol outlet density (units/km^2^) |  |  |  |  |  |  |  |  |  |
| ≤400 m | -0.01 | -0.02, 0.01 | 0.368 | -0.01 | -0.02, 0.01 | 0.247 | -0.01 | -0.02, 0.01 | 0.218 |
| ≤1600 m | 0.02 | -0.22, 0.26 | 0.877 | 0.03 | -0.21, 0.27 | 0.837 | 0.02 | -0.22, 0.26 | 0.842 |
| Distance to the nearest outlet (100 m) | -0.01 | -0.03, 0.01 | 0.495 | -0.01 | -0.02, 0.01 | 0.588 | -0.01 | -0.02, 0.01 | 0.606 |
| **HDL cholesterol** |  |  |  |  |  |  |  |  |  |
| Alcohol outlet density (units/km^2^) |  |  |  |  |  |  |  |  |  |
| ≤400 m | -0.00 | -0.01, 0.00 | 0.802 | -0.00 | -0.01, 0.01 | 0.932 | -0.00 | -0.01, 0.01 | 0.961 |
| ≤1600 m | -0.07 | -0.16, 0.02 | 0.118 | -0.07 | -0.16, 0.02 | 0.109 | -0.07 | -0.16, 0.02 | 0.110 |
| Distance to the nearest outlet (100 m) | 0.00 | -0.00, 0.01 | 0.616 | 0.00 | -0.01, 0.01 | 0.807 | 0.00 | -0.01, 0.01 | 0.819 |
| **LDL cholesterol** |  |  |  |  |  |  |  |  |  |
| Alcohol outlet density (units/km^2^) |  |  |  |  |  |  |  |  |  |
| ≤400 m | -0.00 | -0.01, 0.01 | 0.796 | -0.00 | -0.02, 0.01 | 0.539 | -0.00 | -0.02, 0.01 | 0.502 |
| ≤1600 m | 0.07 | -0.12, 0.26 | 0.476 | 0.07 | -0.12, 0.26 | 0.466 | 0.07 | -0.12, 0.26 | 0.475 |
| Distance to the nearest outlet (100 m) | -0.01 | -0.03, 0.01 | 0.190 | -0.01 | -0.02, 0.01 | 0.310 | -0.01 | -0.02, 0.01 | 0.320 |
| ^*^ independent variable multiplies 1000 to show more information  Model 1 is adjusted for age, sex;  Model 2 is adjusted for model 1+education, occupation, and Standard of Living Index;  Model 3 is adjusted for model 2+tobacco, physical activity, and energy. | | | | | | | | | |
